# Synthesis of Multi-Stimuli Responsive Fe_3_O_4_ Coated with Diamonds Nanocomposite for Magnetic Assisted Chemo-Photothermal Therapy

**DOI:** 10.3390/molecules28041784

**Published:** 2023-02-13

**Authors:** Yang Li, Jichuan Kong, Huan Zhao, Yao Liu

**Affiliations:** 1 School of Medicine, Henan Polytechnic University, Jiaozuo 454000, China; 2 The First Affiliated Hospital, Zhengzhou University, Zhengzhou 450000, China

**Keywords:** nanodiamonds, Fe_3_O_4_, multi-stimuli, magnetic targeting, chemo-photothermal therapy

## Abstract

Nanodiamonds with magnetic resonance imaging (MRI) and targeted drug delivery to exert combined effects for biomedical applications have been considered to be an urgent challenge. Herein, a novel bio-nanoarchitectonics (Fe_3_O_4_@NDs) with simultaneous imaging and therapeutic capacities was fabricated by covalently conjugating nanodiamonds (NDs) with Fe_3_O_4_. Fe_3_O_4_@NDs exhibited better biocompatibility and excellent photothermal stability with superb photothermal conversion performance (37.2%). Fe_3_O_4_@NDs has high doxorubicin (DOX) loading capacity (193 mg/g) with pH and NIR-responsive release characteristics. Fe_3_O_4_@NDs loading DOX showed a combined chemo-photothermal inhibitory effect on the tumor cells. Enhanced T_2_-weighted MRI contrast toward the tumor, with the assistance of a magnetic field, convinced the Fe_3_O_4_@NDs gathered in the tumor more efficiently and could be used for MRI-based cancer diagnosis. Our results revealed an effective strategy to achieve a stimuli-sensitive nanoplatform for multifunctional theranostics by the combined action.

## 1. Introduction

Cancer is the second leading cause of death globally, with an increasing incidence due to the world’s aging population [1]. Although chemotherapy is still one of the dominant methods for clinical cancer treatment, poor tumor accumulation and undesirable side effects reduce the therapeutic effect [2]. Additionally, the premature degradation and rapid renal clearance lead to significant drug resistance and substantial therapy failure [3]. With the advancement of nanotechnology, the strategy of using nanoparticles (NPs) as nano-based drug delivery systems (NDDSs) has been widely investigated for tumor therapy, as they possess considerable potential for targeted delivery of drugs into the tumor cells and protect healthy cells from cytotoxicity of chemotherapeutic drugs [4,5,6]. However, effective retention and successful delivery of anticancer drugs into tumor cells are of equal importance due to drug molecules in the tumor cells will be recognized by transporters (such as P-glycoprotein) and pumped out by tumor cells [7]. Traditional NDDSs face enormous challenges on the drug retention. About 90% of failed tumor treatments are attributed to drug efflux [8]. The most effective solution is to conjugate the drug with specific nanomaterials to form nanocomplexes to escape transporter recognition.

**Scheme 1:**
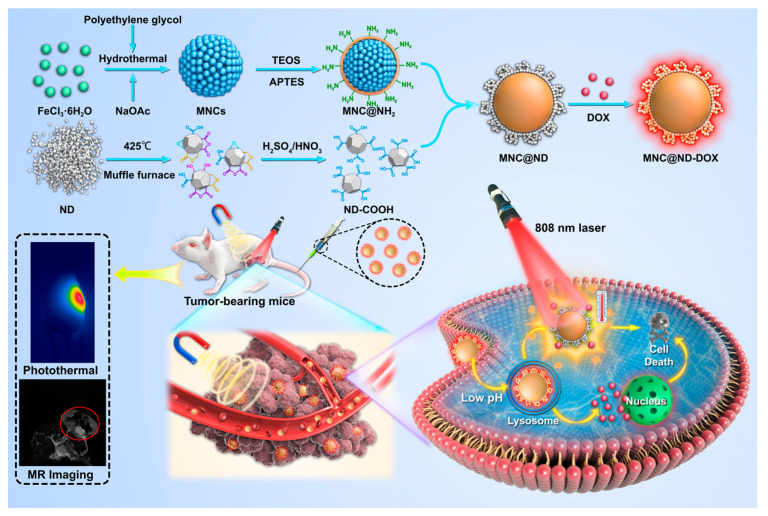
Schematic illustration of the preparation of MNC@ND-DOX and its magnetic targeted and imaging guided combined photothermal/chemotherapy cancer therapy.

Nanodiamond is among the least toxic of all carbon-based nanomaterials tested so far [9,10] and is a potential nanomedicine platform that can bypass the drug efflux mechanisms of tumor cells and enhance intracellular drug retention [8,11]. Recently, drug delivery system-based nanodiamonds (NDs) have attracted growing attention for their superior biocompatibility, chemical stability, and drug accommodating ability [12,13,14,15]. The current targeted delivery of NDs into tumors is based on the enhanced permeability and retention (EPR) effect [12,13,14,15,16]. However, insufficient tumor vascular penetration of such passive accumulation only permits 2–5% of the injected dose into the tumor tissue [17]. Therefore, an effective approach to enhance drug accumulation in the tumor cells is highly desirable for clinical application [18]. To enhance targeting efficiency, active targeted therapy, usually by conjugating with a specific targeting ligand that could selectively bind to their well-defined reaction sites on cancer cells, has become one of the attractive research fields [19,20,21,22]. However, there were slow responses, expensive costs, a complex preparation process, a low accumulation rate at the tumor site, and a lack of response release ability. Moreover, these methods more or less affect the versatile surface chemistry of NDs and decrease drug loading. It may be necessary to apply localized external energy providing physical/mechanical stimuli to assist NDs from the circulation to accumulate into the tumor tissue, such as magnetic field, ultrasound, or heat [23,24,25]. Among them, the magnetic target has been demonstrated to be a promising approach for high-efficacy drug delivery due to its fewer side effects, quick response, and low cost [25,26]. The magnetic target methods have been extensively investigated by researchers in vivo and vitro [27,28,29]. Additionally, it has been used as a magnetic resonance image (MRI) contrast agent for early diagnosis and real-time monitoring tumor therapy [30,31,32]. Thus, endowing ND with magnetism properties is extremely attractive [31,33,34]. In addition, there were few reports on magnetic nanodiamond as a nanodrug delivery system for tumor magnetic targeted therapy so far. In this study, a gentle method for the preparation of multifunctional magnetic nanodiamond-loaded bio-nanoarchitectonics was proposed by efficient amide condensation with F_3_O_4_ NPs (MNC) and NDs to form MNC@NDs. MNC@ND loading chemotherapeutic agents could exhibit high photothermal conversion efficiency and photothermal stability, promising its important role as a photothermal therapy (PTT) agent with the stimuli-responsive release of chemotherapeutic agents. The combination of chemo-photothermal therapy can more effectively kill tumor cells after loading model drug DOX. Under the action of an external MF, MNC@NDs could be enriched more efficiently in the tumor, enabling the MNC@NDs to have active targeting and MR imaging properties (Scheme 1). We hypothesized that MNC@NDs loading DOX could be expected to be an ideal diagnosis and treatment agent for tumors.

## 2. Results and Discussion

### 2.1. Preparation and Characterization of MNCs

**Figure 1:**
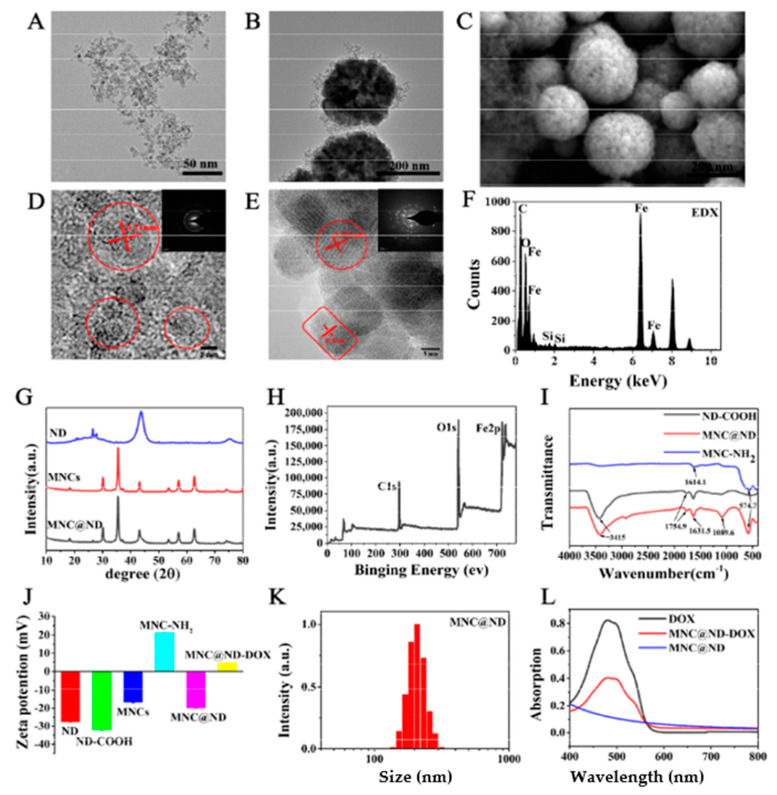
Transmission electron microscopy (TEM) images of (**A**) ND and (**B**) MNC@ND nanoparticles. (**C**) SEM images of MNC@ND. (**D**) HRTEM images of ND nanoparticles, insert is the diffraction pattern of ND NPs. (**E**) HRTEM images of MNCs nanoparticles, insert is the diffraction pattern of MNCs nanoparticles. (**F**) EDX of the MNC@ND. (**G**) XRD patterns of ND, MNC and MNC@ND NPs. (**H**) XPS of MNC@ND NPs. (**I**) FTIR spectrometry of ND-COOH, MNC-NH_2_ and MNC@ND. (**J**) Zeta potential of NDs NPs, ND-COOH, MNC, MNC−NH_2_, MNC@ND and MNC@ND-DOX NPs. (**K**) Size distributions of MNC@NDs. (**L**) UV−visible absorption spectra of DOX, MNC@ND and MNC@ND-DOX NPs.

MNCs were synthesized by hydrothermal method because of the advantages of the hydrothermal method with uniform particle size, good super paramagnetism, and low agglomeration [35]. It was aminated with APTES and followed by amide reaction with carboxylated ND to obtain MNC@NDs. The morphology and characteristics of NDs and MNC@NDs were examined, and as shown in Figure 1A,B, TEM images revealed that ND coated the surface of MNC NPs, forming a core/shell nanostructure. They indicated MNC cores with a diameter of about 120 nm and ND shells with a thickness of about 10–40 nm. A scanning electron microscope (SEM) image of MNC@ND (Figure 1C) showed that MNC@ND NPs were round particles with a size of approximately 150–220 nm with the uniform morphology and good dispersion.

The crystalline structure of the composite shell was shown in Figure 1D. The interplanar distance in the sample area was 0.21 nm, close to the distance of the (110) lattice planes of ND. The HRTEM image showed (111), (220), and (311) lattice planes of the composite shell, confirming that the shell was mainly composed of diamond nanodots. This was further confirmed by XRD (Figure 1G). Three extra diffraction peaks at 26.6°, 43.8°, and 75.4° were attributed to the (111), (220), and (311) planes of the ND cubic phase, respectively. The crystalline structure and electron diffraction pattern of the composite cores were shown in Figure 1E and the interplanar distance in the area of the samples was 0.30 nm, which was close to the distance of the (220) lattice planes of Fe_3_O_4_. The selected area electron diffraction pattern had distinct diffraction rings and arcs that contributed to the classical (220), (311), and (440) reflections of Fe_3_O_4_. XRD measurements on MNCs in Figure 1G showed six diffraction peaks at 30.18°, 35.52°, 43.1°, 53.66°, 57.10°, and 62.82°, which contributed to the (220), (311), (400), (422), (511), and (440) planes of the MNCs cubic inverse spinel phase. To further confirm the contents of MNC@ND, an Energy Dispersive X-Ray Spectrometer (EDX) analysis of the selected area and XPS analysis were conducted, and the results are shown in Figure 1F,H. The EDX result indicated the existence of C, N, O, Fe, and Si elements in MNCs@NDs, and the spectra of XPS showed that there were mainly C, O, and Fe elements in MNCs@NDs. In addition, XPS spectra of Fe2p contained two peaks of Fe2p3/2 and Fe2p1/2, indicating that the valence of iron in MNCs was +2 and +3, which further proved that the main component of the cores was Fe_3_O_4_ nanoparticles (Figure S1).

In order to further explore the assembling mechanism between MNCs and NDs, FTIR and zeta potential analyses were carried out. It can be seen that the extended -OH stretching vibration absorption peak at 3415.37 cm^−1^ on bare NDs and ND-COOH (Figure 1I) The characteristic spectra at 1760.72 cm^−1^ and 1384.66 cm^−1^ demonstrated that the surface of the ND contained abundant C=O and H-O, respectively [36]. The characteristic peaks of H-O disappeared after carboxylation, whereas the characteristic peaks of C=O increased significantly, indicating that the hydroxyl functional groups on the ND surface were oxidized to carboxyl functional groups (Figure S2). Such can be coincident with the zeta potential of the pristine NDs at −28 mV decreasing to −32 mV followed by carboxylated nanodiamonds (Figure 1J). Importantly, a characteristic peak appears at 1089.60 cm^−1^ in the MNC@NDs that could be attributed to the stretching vibration of the C-N bond between NDs and MNCs (Figure 1I and Figure S3), which made the core-shell structure of the MNC@NDs more stable. In addition, the zeta potential values for ND-COOH, MNC-NH2 and MNC@NDs were −28 mV, +20 mV and −25.1 mV respectively. The positive zeta potential of MNCs-NH2 was decreased to −25.1 mV after modified by the NDs, which could contribute to the amidation reaction between NDs and MNCs.

As shown in Figure 1K, the DLS size distributions of MNCs@NDs composite was 150–300 nm ensuring the enhanced permeability and retention effect for nanoscale biomaterials (60–400 nm) and increased the accumulation of nanomaterials at the tumor region [37]. DOX was selected as a model drug to assess the delivery capabilities of MNC@ND. MNC@ND absorbed DOX through electrostatic interaction and formed a stable chemical bond with the carboxyl group on the surface of ND through an amide reaction, which makes DOX for better pH-responsive drug release in the tumor tissue. The UV-Vis absorption spectrum of the MNC@ND-DOX NPs showed a similar absorption peak at 480 nm, indicating successful DOX loading. Due to the positively charged DOX molecules, the zeta potential value of MNCs@NDs NPs increased from −20 to +5.0 mV after DOX loading on MNCs@NDs NPs also showed successful DOX loading.

### 2.2. DOX Loading and In Vitro Release

**Figure 2:**
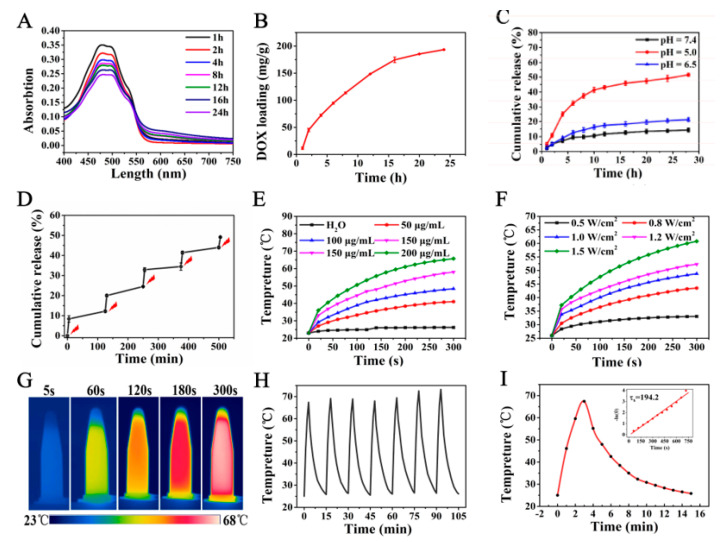
(**A**) UV−visible absorption spectra of DOX in the supernatant at different time. (**B**) Plot of DOX loading amount for MNC@ND-DOX versus different time. (**C**) DOX release from MNC@ND-DOX NPs in buffers at the different pH values (5.0, 6.5 and 7.4). (**D**) DOX release from MNC@ND-DOX NPs at the NIR irradiation (pH = 5.0). (**E**) Temperature change curves of MNC@ND solutions with different concentrations under 808 nm NIR laser irradiation (1 W/cm^2^, 5 min) (**F**) Effects of varying laser−irradiation power at MNC@ND-DOX NPs concentration of 100 μg/mL are shown. (**G**) Photothermal imaging of varying time of MNC@ND-DOX NPs (200 μg/mL) under laser−irradiation. (**H**) The photothermal stability of MNC@ND-DOX solution (200 μg/mL) irradiated under an 808 nm laser at 1.0 W/cm^2^ for 300 s, then the laser was turned off. (**I**) The photothermal response of MNC@ND solution irradiated under an 808 nm laser at 1.5 W/cm^2^ for 180 s, then the laser was turned off, insert is linearity curves fitted from the temperature cooling time vs −ln(θ) of MNC@ND (200 μg/mL 1.0 W/cm^2^).

The amount of DOX loaded into the MNCs@NDs NPs was estimated by measuring the absorbance at 480 nm and calculated by the standard curves equation of DOX (Figure S4). As shown in Figure 2A, the absorbance of the DOX in supernatant at 480 nm decreased gradually with increasing time, suggesting that the amount of DOX loaded on the MNCs@NDs NPs increased over time. About 38.6% (193 mg/g) of DOX was eventually loaded into MNCs@NDs, and the results were shown in Figure 2B. The high drug loading capacity may be attributed to the rough structure on the surface of the ND shell, and aromatic DOX had a strong interaction with the ND materials via supramolecular π-stacking [7]. In addition, the negative charges of the ND surface and abundant carboxyl groups had strong electrostatic interaction with DOX. To examine the internal stimulus-response release of DOX, the MNCs@ND-DOX was placed in phosphate buffer saline (PBS) with various pH values (pH = 5.0, 6.5, and 7.4) and the drug release profiles of MNCs@ND-DOX were shown in Figure 2C. When the pH value of the solution was 7.4 and 6.5, the release rate of DOX was very slow. Only 14.7% and 21.5% DOX were released. While accelerated release was observed at pH 5.0, and the release content was as high as 53%. Additionally, NIR pulse irradiation for the detection ofMNCs@ND-DOX drug release was carried out at pH 5.0, as shown in Figure 2D, upon the irradiation of NIR light; a significant drug burst release was observed, and the burst released phenomenon was attributed to the local thermal energy produced by MNCs under NIR light irradiation, resulting in the dissociation of DOX from MNCs@ND-DOX. The pH and NIR-responsive nanocarrier favored using in the practical biomedical field due to the pH range in the tumor intracellular environment being 5.0–5.5. The lower drug release at pH = 7.4 physiological conditions could reduce the adverse effects on normal organs. Compared with mono stimuli-responsive drug delivery systems, double stimuli-responsive drug delivery systems could realize a more efficient and on-demand drug release in tumor tissues and improve chemotherapy efficacy.

### 2.3. In Vitro Photothermal Effect

The photothermal performance of the MNCs@ND was explored in detail. The temperature changes of aqueous solutions containing different concentrations of MNCs@ND exposed to an 808 nm NIR laser (1.0 W/cm^2^, 5 min) were shown in Figure 2E. Upon 808-nm NIR laser irradiation, the temperature increases of the sample solutions with concentrations of 50, 100, 150, and 200 μg/mL were 18.0 °C, 25.4 °C, 35.0 °C, and 42.7 °C, respectively. Assuming that the normal human body temperature was 37 °C, tumor tissues could be easily heated to the temperature of hyperthermic therapy after injecting with MNCs@ND-DOX NPs, even at a concentration as low as 50 μg/mL, and there was a concentration-effect relationship between concentration and temperature. The results demonstrated that the MNCs@ND NPs could effectively transform NIR light into heat energy commendably, making them potential for tumor PTT.

The temperature changes of aqueous solutions containing 100 μg/mL of MNCs@NDs exposed to different power of 808 nm NIR laser (0.5, 0.8, 1.0, 1.2 and 1.5 W/cm^2^) were shown in Figure 2F. The temperature of MNCs@NDs (100 μg/mL) increased rapidly with the increase of laser intensity and the temperature reached over 50 °C under 1.0 W/cm^2^, which is enough to photothermal therapy. The photothermal photographs of MNC@ND in vitro were investigated by an infrared thermal camera, and the results were shown in Figure 2G. With the same concentration, the temperature of the MNC@ND solution containing 200 μg/mL gradually increases to 67.6 °C. Furthermore, the photothermal stability test of MNCs@ND was conducted and the results were shown in Figure 2H. The results of the tests showed that the photothermal conversion ability of the MNCs@ND did not decrease even after seven photothermal cycles. The photothermal conversion efficiency (η) was calculated to be 37.2% by the formula based on previous literature (see calculation formula in Supporting Information), as shown in Figure 2I. The high photothermal conversion efficiency of the MNCs@ND-DOX implied that it could be used for the photothermal therapy of a tumor.

### 2.4. Cellular Uptake

**Figure 3:**
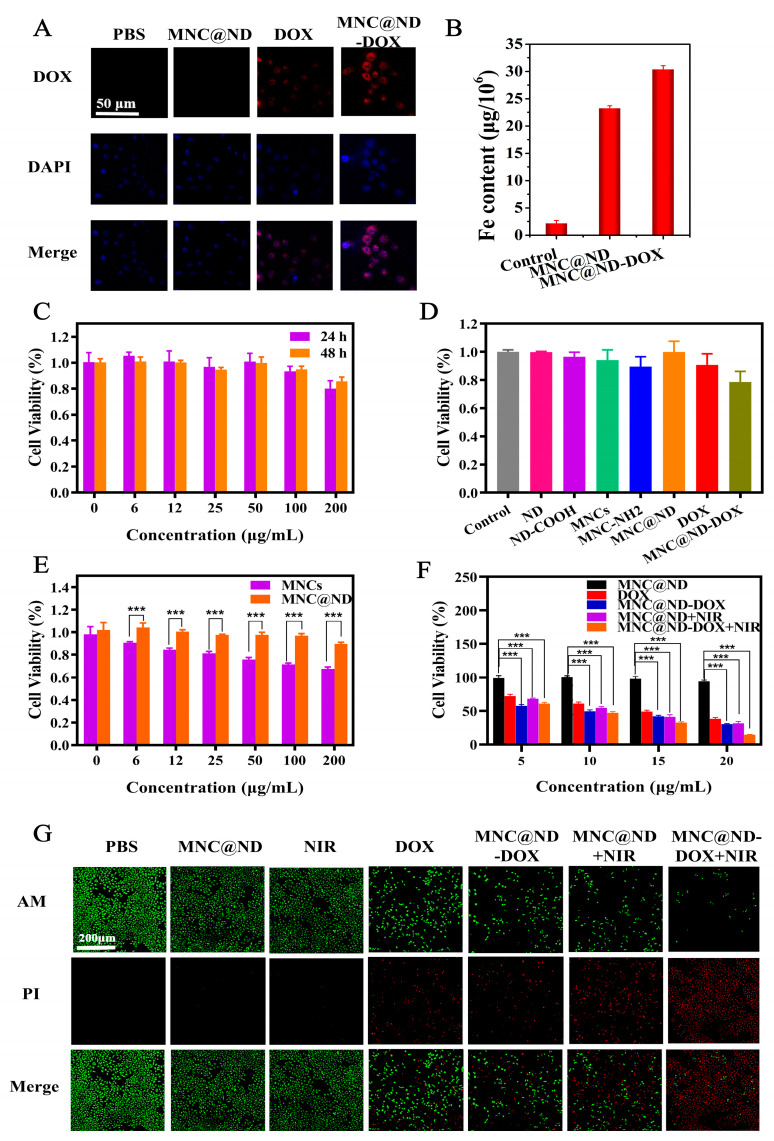
(**A**) Confocal fluorescence images of A549 cells incubated with PBS, MNC@ND NPs, DOX and MNC@ND-DOX NPs for 4 h. (**B**) The amount of Fe element in A549 cell. (**C**) Cell viability of A549 cells treated with different concentration of MNC@ND NPs after 24 and 48 h. (**D**) Cell viability of A549 cells treated with different materials. (**E**) Cell viability of A549 cells treated with different concentration of MNCs and MNCs@ND, A549 cells treated with 0 concentration were used as control. (**F**) Cell viability of A549 cells treated with MNC@ND NPs, DOX, MNC@ND-DOX NPs, MNC@ND NPs + NIR and MNC@ND-DOX NPs+NIR at different concentrations of DOX and the corresponding amount of drug-free nanocapsules. Data are mean ± SD (*n* = 3, *** *p* < 0.001). (**G**) Fluorescence images of calcein AM/PI-stained A549 cells incubated with PBS, MNC@ND, NIR, DOX, MNC@ND-DOX NPs, MNC@ND+NIR and MNC@ND-DOX+NIR NPs.

The cellular uptake behaviors of MNC@ND-DOX inside A549 cells were carried out by a confocal laser scanning microscopy (CLSM) observation and Inductively Coupled Plasma Mass Spectrometry (ICP-MS). As shown in Figure 3A, after A549 cells incubating with PBS, MNC@ND, DOX and MNC@ND-DOX, red fluorescence was apparently ob-served in the cytoplasm under the DOX excitation pathway, while only blue fluorescence in DAPI mode was observed in PBS and MNC@ND groups, indicating that free DOX and MNC@ND-DOX had been uptaken by the A549 cells. The fluorescence images could show MNC@ND-DOX delivered DOX into A549 cells more efficiently. To further confirm the MNC@ND-DOX internalization in cells, the context of Fe was tested by ICP-MS; as shown in Figure 3B, 15.1 and 17.1 μg Fe element per 10^6^ A549 cells treated with MNC@ND and MNC@ND-DOX for 24 h were found, respectively, while only 1.41 μg Fe element in blank cells was found. All the above indicated that MNC@ND and MNC@ND-DOX can be transported into cells, and MNC@ND-DOX could release DOX to inhibit cell growth.

### 2.5. Biocompatibility and Cytotoxicity 

The biocompatibility of the nanoparticles was an important issue in the fabrication of drug delivery systems. The CCK-8 cytotoxicity test was used to value the compatibility and therapeutic properties of MNC@ND-DOX, and the results are shown in Figure 3C. The cell viability of A549 cells incubated with MNC@ND did not obviously decrease over 24 and 48 h when the concentration was increased from 0 to 200 μg/mL, indicating that MNC@ND had good cell biocompatibility. The biocompatibility of NDs, ND-COOH, MNCs, MNC-NH_2_, and MNC@ND was also evaluated in vitro using a CCK-8 assay, and the results were shown in Figure 3D. Note that when MNCs were modified with NDs to form a MNC@ND composite structure, their biocompatibility improved. Compared with DOX, MNC@ND-DOX killed more cancer cells, and the results were consistent with those of confocal fluorescence images (Figure 3A). This indicated that DOX could be successfully conducted by MNC@ND and continuously released from MNC@ND-DOX to suppress tumor cells.

In general, MNC could damage the cellular organelles, such as lysosomes, mitochondria, and endoplasmic reticulum, and improved the autophagy level leading to cell death [38]. Nevertheless, it has been found that appropriate surface modification could significantly reduce these untoward effects [39]. As shown in Figure 3E, A549 cells treated with various concentrations of MNCs showed a dose-dependent cytotoxicity. In contrast, when the MNCs was coated with nanodiamonds particles, the cell viability increased significantly (*p* < 0.001). Thus, the modification of nanodiamonds not only could improve the drug loading but also could further reduce the toxicity and enhance the biocompatibility of nanocomposites. 

In order to explore the chemotherapy effect of MNC@ND-DOX, we compared the tumor cell viability of MNC@ND-DOX with DOX (contains an equal amount of DOX) and the results were shown in Figure 3F. The cell viability of A549 significantly (*p* < 0.001) decreased with the increasing concentration of MNC@ND-DOX. The results indicated that MNC@ND-DOX exhibited a good chemotherapy effect. The photothermal therapeutic effect of MNC@ND was evaluated under a 808 nm laser (1.0 W/cm^2^, 5 min), and the results showed that the viability of A549 incubated with MNC@ND obviously reduced, indicating that MNC@ND NPs had a good PTT. The chemo-photothermal combination therapy effect of MNC@ND-DOX NPs in vitro was also investigated by CCK-8 assay, and the results are shown in Figure 3F. The lowest cell viabilities were achieved when cells were incubated with MNC@ND-DOX under 808 nm light exposure, indicating the combination of photothermal and chemotherapy could possess an excellent therapeutic effect on cancer cells.

Calcein AM (green) and PI (red) co-staining assays were carried out to further verify the anti-tumor effect of MNC@ND-DOX in vitro due to it could distinguish between live and dead cells. The results shownin Figure 3G, the green fluorescence in the control, and the MNC@ND and laser groups indicated that the MNC@ND and laser alone were nontoxic to tumor cells. When incubated with DOX and MNC@ND-DOX, the intense red fluorescence demonstrated that the death rate of cells increased because DOX entered the cell’s nucleus to further destroy the DNA structure. A bigger field of red fluorescence of A549 cells incubated with MNC@ND under NIR laser irradiation denoted that MNC@ND effectively killed tumor cells for the photothermal effects. More importantly, when cancer cells were incubated with MNC@ND-DOX NPs under NIR laser irradiation, more intense red fluorescence implied that the cell death rate increased. These results came from the fact that upon NIR laser irradiation, MNC@ND-DOX NPs constantly released DOX and gathered hyperthermia, allowing chemo-photothermal combination therapy to efficiently kill tumor cells.

### 2.6. In Vivo MR Imaging

**Figure 4:**
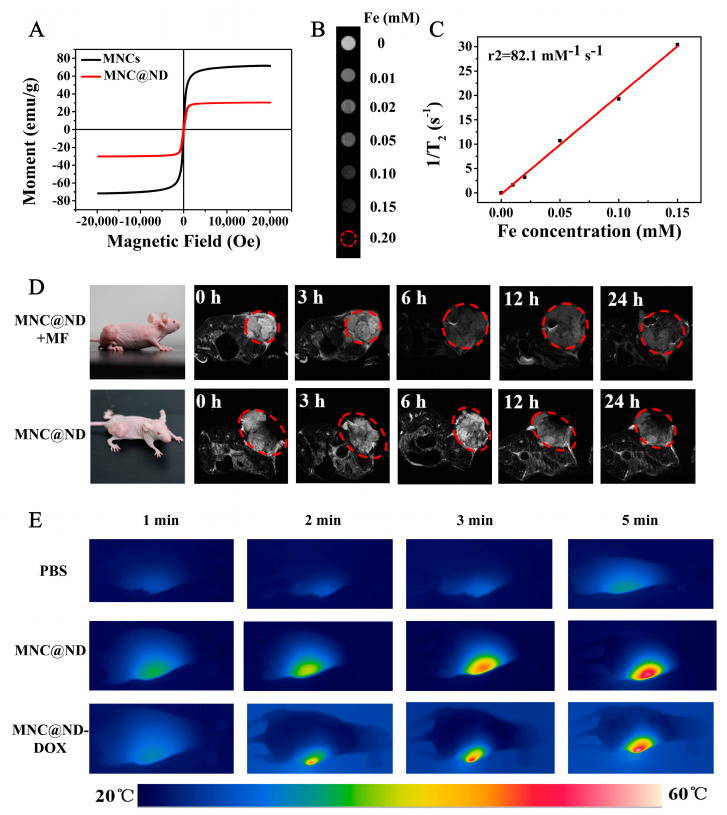
(**A**) Magnetization curve of MNCs and MNCs@ND. (**B**) T_2_−Weighted MR imaging of MNC@ND in PBS buffer (pH 6.0) incubated for different periods of time. (**C**) T_2_ relaxation rates (r_2_) in the case MNC@ND-DOX at different concentrations. (**D**) In vivo T_2_-weighted MR images of a tumor-bearing mouse taken before and after injection at 0, 3, 6, 12 and 24 h of MNC@ND-DOX (14.26 mg/kg). The tumor regions are pointed out in the red circle. (**E**) Infrared thermal images of tumor-bearing mice treated with PBS, MNC@ND, and MNC@ND-DOX via irradiation with 808 nm NIR light for different time periods.

The MNC@ND had a big magnetization saturation value of 30 emu/g (Figure 4A). This indicated that the MNC@ND had good magnetic properties and could be used as an ideal T_2_-weighted MRI agent. In vitro T_2_-weighted MRI of MNC@ND NPs was shown in the Figure 4B. With the increasing of iron concentration, the T_2_ signal intensity increased gradually, indicating a concentration-dependent darkening field effect. As shown in Figure 4C, the transverse relaxivity (r_2_) of the MNC@ND was measured to be 14.322 mM^−1^ s^−1^. The tumor-targeting ability of MNC@ND-DOX on tumor-bearing mice was investigated by MRI, and the T_2_-weighted MR images of the tumor-bearing mice treated at different time intervals (0, 3, 6, 12, and 24 h) were shown in Figure 4D. MNC@ND-DOX entered the blood circulation after injection, and some NPs were gradually deposited on the tumor site due to the EPR effect; a significant darkening effect in the tumor region of A549 tumor-bearing mouse could be easily detected after 12 h. While under the assistance of magnetic field, T_2_-weighted images at the tumor region showed obvious darken image within 6h, demonstrating that the well-designed MNC@ND could more efficiently accumulate into tumors and possessed the stronger ability to target tumors upon the help of external magnetic field. These results indicated MNC@ND NPs had excellent magnetic assisted targeting capability and could be used as MR imaging-guided antitumor theranostic reagents.

### 2.7. In Vivo Photothermal Effect

The photothermal effect of MNC@ND-DOX in vivo was investigated by an infrared thermal camera. Mice bearing A549 tumors were intravenously injected with MNC@ND-DOX and MNC@ND and exposed to an 808 nm laser (1.0 W/cm^2^ for 5 min) at 24 h post-injection. It can be seen in Figure 4E that the temperature of tumor region rapidly increased to 54.3 °C and 57.4 °C, respectively. By comparison, tumor temperature for mice treated with PBS only was elevated by 4.3 °C. The results showed that MNC@ND-DOX NPs could be used as an excellent photothermal agent for high-efficiently photothermal treatment of tumor in vivo.

## 3. Materials and Methods

### 3.1. Materials

All chemicals and reagents were used without further purification unless otherwise noted. The nanodiamonds were purchased from Sino Crytal Micro Diamond Co. Ltd. (Zhengzhou, China). Ferric chloride hexahydrate (FeCl_3_·6H_2_O), morpholine ethyl sulfonic acid (MES), ethylene glycol (CH_2_OHCH_2_OH), polyethylene glycol, (3-aminopropyl)-trimethoxy silane (APTES), and sodium acetate (CH_3_COONa) were purchased from Sigma-Aldrich (Shanghai, China) and used as received. Doxorubicin hydrochloride (DOX·HCl) was supplied by Shanghai Jinhe Bio-Technology Co. Ltd (Shanghai, China). Dulbecco’s minimum essential medium (DMEM) cell culture medium, penicillin/streptomycin (PS), and fetal bovine serum (FBS) were bought from Gibco Invitrogen (New York, USA). Human lung adenocarcinoma A549 cells were purchased from the Chinese Academy of Sciences Cell Bank. Cell Counting Kit-8 (CCK-8 assay), 4′,6-diamidino-2-phenylindole (DAPI), Calcein-AM and propidium iodide (PI) were purchased from Summus (Zhengzhou, China). Deionized (DI) water was freshly obtained using the Milli-Q water purification system (Millipore, Bedford, MA, USA).

### 3.2. Celland Animals Model

Human lung adenocarcinoma A549 cells were purchased from the Chinese Academy of Sciences Cell Bank and cultured in DMEM medium (Gibco, New York, USA) supplemented with 10% fetal bovine serum and 1% penicillin/streptomycin in a humidified atmosphere at 37 °C under 5% CO_2_. Female nude BALB/c 4 week-old mice were purchased from Beijing Sibafu Biotechnology Co. LTD (Beijing, China). Animals were fed with standard conditions. Female nude mice (20 g) were inoculated by subcutaneous injection of 5 × 10^6^ cells in 200 μL PBS on the flank of each mouse. All the experimental steps adopted in this experiment conformed to the experimental scheme approved by Animal Experiment Center of Zhengzhou University. All animal operations were carried out in accordance with the guiding principles of the “Declaration of Helsinki, DoH” and approved by the Medical Ethics Committee (2021042501) of the First Affiliated Hospital of Zheng Zhou University. 

### 3.3. Preparation of ND-COOH

The nanodiamond powder, having particle size of <10 nm, was carboxylated by a modified standard procedure [40]. Primarily, the obtained nanodiamond powder was calcined in a muffle furnace for 5 h at 425 °C and then cooled to room temperature; the obtained nanodiamond powder was then treated with the mixture of H_2_SO_4_ and HNO_3_ (3:1 *v*/*v*) at 90 °C for 48 h, diluted with deionized water, and centrifuged at 8000 rpm for 15 min to obtain the ND particles. The obtained particles were rinsed by deionized H_2_O, which was further heated at 90 °C for 2 h in 0.1 M NaOH solution. Then, ND sample was heated again with 0.1 M HCl for 2 h at 90 °C and washed twice by deionized water until the solution became weakly acidic. The obtained carboxylated-NDs were separated and dried under vacuum for further procedure.

### 3.4. Preparation of MNCs Nanoparticles

The high-quality magnetic nanoparticles were synthesized by a modified hydrothermal method [35]. Typically, 1.35 g of FeCl_3_·6H_2_O was added to 40 mL of ethylene glycol and sonicated for 5 min. The mixture was stirred to completely dissolve it; 3.6 g of NaOAc and 0.5 g of polyethylene glycol (molecular weight 6000) were added into the reaction system and stirred vigorously for another 5 min, then transferred into a Teflon-lined stainless-steel auto-clave, followed by heating at 200 °C for 20 h. The products were magnetically separated and washed three times with DI water and ethanol. The obtained MNCs were dried at 50 °C under vacuum for further procedure.

### 3.5. Preparation of MNCs@SiO_2_-NH_2_ Nanoparticles

MNCs@SiO_2_-NH_2_ nanoparticles were synthesized by a reversed-phase microemulsion method according to methods previously reported [41]. A total of 750 mg of magnetic nanoparticle powder was dissolved in a mixing solution of 100 mL deionized water and 50 mL ethanol and sonicated for 40 min until a transparent reversed-phase microemulsion was formed. After that, transparent reversed-phase microemulsion was mechanically stirred in a 300 mL three-necked flask bubbled with nitrogen air at 60 °C for 40 min. Then, dilute hydrochloric acid (0.001 mol/L) was added to adjust the pH of the reaction system to 4.0. After that, 5 mL NH_3_·H_2_O (15 wt%) was added into the solution, and the container was sealed and vigorously stirred for 10 min. Then, 20 mL TEOS was added into the solution and 2 mL APTES was dropped into the mixture after 2 h. After continuous stirring at 50 °C for an additional 4 h, products were precipitated, washed with ethanol several times, and dried at 35 °C under vacuum.

### 3.6. Synthesis of MNCs@NDs

A total of 200 mg of carboxylated nanodiamonds was dispersed into 300 mL morolineethanesulfonic acid buffer solution (pH = 6.0) and sonicated for 10 min; 0.1 g EDC and 0.2 g NHS were added into the solution and stirred for 20 min to activate the carboxyl groups. Subsequently, the nanodiamonds were centrifuged out and re-dispersed into phosphate buffer solution (pH = 7.4). A total of 100 mg of MNCs@SiO_2_-NH_2_ was added to the solution and stirred at room temperature for 12 h. Then, the final product was collected by magnetic separation, washed with deionized water several times, and dried at 35 °C under vacuum.

### 3.7. Characterization

The morphology of the MNC@NDs was tested by HITA-CHIH-7650 transmission electron microscopy (TEM) and ZEISS Gemini SEM 300 scanning electron microscopy (SEM). The ζ-potential and size distribution were determined by Zeta-Sizer, (CA, USA). X-ray diffraction (XRD) patterns were obtained by a Rigaku Ultima IV powder diffractometer. The material composition was determined by X-ray photoelectron spectroscopy (XPS) analysis (E250, Thermo-Fisher Scientific, MA, USA). UV-vis spectra were measured by UV-vis spectrophotometer (UV-2600). The Fourier transform infrared (FTIR) spectra of samples were detected by a NEXUS670 FTIR spectrometer. Magnetic properties of samples were analyzed under an applied magnetic field by a vibrating sample magnetometer (HH-15) at room temperature (Squid). FL images were acquired by a confocal laser scanning microscope (CLSM) (TCSSP5, Leica, Germany). An infrared camera (Fluke TiR) was used to obtain thermal images. The 808 nm laser light source was an LSR808NL-2 W semiconductor power-tunable laser. The MR imaging was acquired on a 4.7 T small animal MR scanner (Bruker I-CON). 

### 3.8. DOX Loading

A total of 10 mg of MNCs@NDs was added into 30 mL of phosphate-buffered saline (PBS, pH = 7.4) containing 0.17 mg/mL of DOX under stirring for 24 h. Unloaded DOX was removed by centrifugation, and MNCs@ND-DOX was washed with PBS and dried under vacuum. The amount of unloaded DOX was measured in the supernatant by UV−vis spectrophotometer at 480 nm. The DOX loading content in the MNCs@ND was calculated by [(M_1_ − M_2_)/M_3_] × 100%, where M_1_ represented the original weight of DOX added, M_2_ was the weight of DOX in the supernatant, and M_3_ was the weight of MNCs@ND-DOX.

### 3.9. Stimuli-Responsive DOX Release from MNCs@ND-DOX

Briefly, 5 mL of PBS containing MNCs@ND/DOX was added to a dialysis tube [molecular weight cut-off (MWCO) = 14,000 Da] immersed in 50 mL release media under gentle stirring at 37 °C. Release medium (2 mL) was collected at the given time intervals, and 2 mL of fresh media was added to the solution. The collected solution was measured the concentration of DOX via a UV-Vis spectrophotometer at 480 nm. The NIR-responsive DOX release of MNCs@ND-DOX was evaluated as follows: MNCs@ND-DOX was dispersed in 5 mL of PBS (pH = 5.0). The mixture was transferred to a dialysis tube (MWCO = 14,000 Da) and immersed in 50 mL of release media under gentle stirring at 37 °C. At given time intervals, the dialysis tube wasirradiated by NIR light (808 nm, 1.0 W/cm^2^) for 300 s, and then 2 mL of the release medium was collected, and equal volume of fresh media was supplied. The collected samples were used to measure the DOX concentration.

### 3.10. Photothermal Performance of MNCs@ND

Different concentrations (0, 50, 100, 150, and 200 μg/mL) of MNCs@NDs NPs were deposited into quartz tubes and then subjected to an 808 nm continuous-wave laser at a power density of 0.5 W/cm^2^, 0.8 W/cm^2^, 1.0 W/cm^2^, 1.2 W/cm^2^, and 1.5 W/cm^2^ for 5 min. A thermocouple probe with an accuracy of 0.1 °C was inserted into the aqueous solution to measure the temperature change. The photo-thermal stability of MNCs@NDs NPs was also examined for 7 cycles. The photothermal conversion efficiency (η) was calculated according to the formula shown in Supporting Information.

### 3.11. Cellar Uptake 

The cellular uptake of MNCs@ND-DOX NPs was investigated by a confocal laser scanning microscope (CLSM). Briefly, A549 cells were seeded at a density of 1 × 10^5^ cells/well on 35 mm Petri dish and incubated at 37 °C for 12 h. Then, MNCs@ND-DOX NPs solution was diluted to 50 μg/mL by DMEM. The cells were treated with MNCs@ND, DOX or MNCs@ND-DOX (containing 10 µg/mL DOX) for another 4 h at 37 °C, rinsed with PBS, fixed with 4% paraformaldehyde, processed with DAPI for 20 min for nuclei staining, and observed by using confocal laser scanning microscope. In order to evaluate the cellular uptake of the MNCs@ND-DOX and MNCs@ND, A549 cells were seeded at a density of 1 × 10^6^ cells/well on 6-well plants and were treated with MNCs@ND or MNCs@ND-DOX for 4 h at 37 °C. After washing with PBS, the cells were segregated to measure the accumulation of Fe content in A549 cells by inductively coupled plasma-mass spectroscopy (ICP-MS, PerkinElmer).

### 3.12. In Vitro Cytotoxicity Assay

In vitro cytotoxicity was measured by CCK-8 assay on A549 cells. The A549 cells were seeded at a density of 5 × 10^3^ cells/well on 96-well plants and incubated with different concentrations of MNCs@NDs, DOX, and MNCs@ND-DOX for 24 h. Then, CCK-8 solution (10 μL, 2 mg/mL) was added into each well. The OD value at 450 nm was tested after 2 h. To evaluate the PTT effect of MNCs@NDs and MNCs@ND-DOX, the cells were incubated with MNCs@NDs and MNCs@ND-DOX for 2 h, followed by irradiating with an 808 nm laser at 1.0 W/cm^2^ for 5 min, then incubated for another 22 h. We measured cell viabilities by the standard CCK-8 assay.

### 3.13. In Vitro Living-Dead Staining

The A549 cells were seed at a density of 5 × 10^5^ cells/well on 6-well plants and were co-incubated with 2 mL different materials medium for 24 h, irradiated with 808 nm laser for 5 min (1.0 W/cm^2^), and stained with calcein-AM and PI. The stained cells were immediately measured by a confocal laser scanning microscope.

### 3.14. In Vivo MR Imaging

For the imaging test, the tumor-bearing mice were injected with MNCs@ND-DOX (12.4 mg/kg) via the tail vein. Then, an external magnetic field was applied to the tumor region for 2 h. T_2_-weighted images were acquired using the 4.7 T clinical MRI scanner (Bruker Icon, Karlsruhe, Germany).

### 3.15. In Vivo Photothermal Imaging

The A549 tumor-bearing mice were intravenously injected with MNCs@ND-DOX (2.48 mg/mL, 100 µL), and then an external magnetic field was applied to the tumor region for 2 h. Then, the tumor area was irradiated by the 808 nm laser at power density of 1.0 W/cm^2^ for 5 min. The control groups of mice were administered with saline and subjected to laser irradiation (MNCs@NDs and PBS). Thermal imaging was recorded by a PTT monitoring MG33 system when the tumors were exposed to the 808 nm laser at power density of 1.0 W/cm^2^ for 5 min.

## 4. Conclusions

In this study, we encapsulated Fe_3_O_4_ (MNCs) with ND to obtain MNC@ND. It had excellent biocompatibility, high photothermal conversion efficiency, excellent photothermal stability, and high doxorubicin (DOX) loading capacity with pH and NIR-responsive release characteristics. MRI on tumor-bearing mice was performed using intrinsic physical and natural MNCs, demonstrating the efficient accumulation of NPs at tumor sites via magnetic assistance. MNC@ND loading DOX possessed good photothermal effects and enhanced DOX release under IR laser irradiation. Tumor cells were effectively inhibited through combination chemo-photothermal therapy with no obvious toxicity. Such MNC@ND-DOX could be a promising nanoplatform for MR and photothermal imaging-guided highly effective chemo-photothermal treatment for cancer.

## Data Availability

Not applicable.

## Supplementary Materials

The following supporting information can be downloaded at: https://www.mdpi.com/article/10.3390/molecules28041784/s1, Figure S1: The XPS spectra of C1s(A), O1s(B), Fe2p(C); Figure S2: FTIR spectrometry of ND and ND-COOH. Figure S3: FTIR spectrometry of MNC@ND. Figure S4: The standard curves of UV-vis absorption values of DOX.

## References

[1] Sung H., Ferlay J., Siegel R.L., Laversanne M., Soerjomataram I., Jemal A., Bray F. (2021). Global cancer statistics 2020: Globocan estimates of incidence and mortality worldwide for 36 cancers in 185 countries. CA Cancer J. Clin..

[2] Dagogo-Jack I., Shaw A.T. (2018). Tumour heterogeneity and resistance to cancer therapies. Nat. Rev. Clin. Oncol..

[3] Dewhirst M.W., Secomb T.W. (2017). Transport of drugs from blood vessels to tumour tissue. Nat. Rev. Cancer.

[4] Rebanda M.M., Bettini S., Blasi L., Gaballo A., Ragusa A., Quarta A., Piccirillo C. (2022). Poly(l-lactide-co-caprolactone-co-glycolide)-based nanoparticles as delivery platform: Effect of the surfactants on characteristics and delivery efficiency. Nanomaterials.

[5] Wang M., Liang Y., Zhang Z., Ren G., Liu Y., Wu S., Shen J. (2019). Ag@Fe_3_O_4_@c nanoparticles for multi-modal imaging-guided chemo-photothermal synergistic targeting for cancer therapy. Anal. Chim. Acta.

[6] Wu F., Zhang M., Lu H., Liang D., Huang Y., Xia Y., Hu Y., Hu S., Wang J., Yi X. (2018). Triple stimuli-responsive magnetic hollow porous carbon-based nanodrug delivery system for magnetic resonance imaging-guided synergistic photothermal/chemotherapy of cancer. ACS Appl. Mater. Interfaces.

[7] Wang X., Low X.C., Hou W., Abdullah L.N., Toh T.B., Mohd Abdul Rashid M., Ho D., Chow E.K. (2014). Epirubicin-adsorbed nanodiamonds kill chemoresistant hepatic cancer stem cells. ACS Nano.

[8] Whitlow J., Pacelli S., Paul A. (2017). Multifunctional nanodiamonds in regenerative medicine: Recent advances and future directions. J. Control. Release.

[9] Moore L., Yang J., Lan T.T.H., Osawa E., Lee D., Johnson W.D., Xi J., Chow E.K., Ho D. (2016). Biocompatibility assessment of detonation nanodiamond in non-human primates and rats using histological, hematologic, and urine analysis. ACS Nano.

[10] Raja I.S., Song S., Kang M.S., Lee Y.B., Kim B., Hong S.W., Jeong S.J., Lee J., Han D. (2019). Toxicity of zero- and one-dimensional carbon nanomaterials. Nanomaterials.

[11] Basu S., Pacelli S., Wang J., Paul A. (2017). Adoption of nanodiamonds as biomedical materials for bone repair. Nanomedicine.

[12] Jung H.S., Neuman K.C. (2021). Surface modification of fluorescent nanodiamonds for biological applications. Nanomaterials.

[13] Liu C., Lee M., Lin H., Lin Y., Lai W., Chien Y., Huo T., Lo W., Lan Y., Chen Y. (2021). Nanodiamond-based microrna delivery system promotes pluripotent stem cells toward myocardiogenic reprogramming. J. Chin. Med. Assoc..

[14] Long W., Ouyang H., Wan W., Yan W., Zhou C., Huang H., Liu M., Zhang X., Feng Y., Wei Y. (2020). “two in one”: Simultaneous functionalization and dox loading for fabrication of nanodiamond-based ph responsive drug delivery system. Mater. Sci. Eng. C.

[15] Pandey P.C., Shukla S., Pandey G., Narayan R.J. (2021). Nanostructured diamond for biomedical applications. Nanotechnology.

[16] Maeda H. (2001). The enhanced permeability and retention (epr) effect in tumor vasculature: The key role of tumor-selective macromolecular drug targeting. Adv. Enzym. Regul..

[17] Mo S., Carlisle R., Laga R., Myers R., Graham S., Cawood R., Ulbrich K., Seymour L., Coussios C. (2015). Increasing the density of nanomedicines improves their ultrasound-mediated delivery to tumours. J. Control. Release.

[18] Park K. (2013). Facing the truth about nanotechnology in drug delivery. ACS Nano.

[19] Li D., Chen X., Wang H., Liu J., Zheng M., Fu Y., Yu Y., Zhi J. (2017). Cetuximab-conjugated nanodiamonds drug delivery system for enhanced targeting therapy and 3d raman imaging. J. Biophotonics.

[20] Liao W., Ho Y., Lin Y., Naveen Raj E., Liu K., Chen C., Zhou X., Lu K., Chao J. (2019). Targeting egfr of triple-negative breast cancer enhances the therapeutic efficacy of paclitaxel- and cetuximab-conjugated nanodiamond nanocomposite. Acta Biomater..

[21] Slegerova J., Hajek M., Rehor I., Sedlak F., Stursa J., Hruby M., Cigler P. (2015). Designing the nanobiointerface of fluorescent nanodiamonds: Highly selective targeting of glioma cancer cells. Nanoscale.

[22] Zhang X.Q., Lam R., Xu X., Chow E.K., Kim H.J., Ho D. (2011). Multimodal nanodiamond drug delivery carriers for selective targeting, imaging, and enhanced chemotherapeutic efficacy. Adv. Mater..

[23] Li H., Ma M., Zhang J., Hou W., Chen H., Zeng D., Wang Z. (2019). Ultrasound-enhanced delivery of doxorubicin-loaded nanodiamonds from pullulan-all-trans-retinal nanoparticles for effective cancer therapy. ACS Appl. Mater. Interfaces.

[24] Li H., Zeng D., Wang Z., Fang L., Li F., Wang Z. (2018). Ultrasound-enhanced delivery of doxorubicin/all-trans retinoic acid-loaded nanodiamonds into tumors. Nanomedicine.

[25] Saadat M., Manshadi M.K.D., Mohammadi M., Zare M.J., Zarei M., Kamali R., Sanati-Nezhad A. (2020). Magnetic particle targeting for diagnosis and therapy of lung cancers. J. Control. Release.

[26] Wang J., Zhu F., Li K., Xu J., Li P., Fan Y. (2022). Ph-responsive mesoporous Fe_2_O_3_–au nanomedicine delivery system with magnetic targeting for cancer therapy. Med. Nov. Technol. Devices.

[27] Bai C., Hu P., Liu D., Chen Y., Ma M., Gu N., Zhang Y. (2020). A novel method to construct dual-targeted magnetic nanoprobes by modular assembling. Colloids Surf. A Physicochem. Eng. Asp..

[28] Mayorova O.A., Sindeeva O.A., Lomova M.V., Gusliakova O.I., Tarakanchikova Y.V., Tyutyaev E.V., Pinyaev S.I., Kulikov O.A., German S.V., Pyataev N.A. (2020). Endovascular addressing improves the effectiveness of magnetic targeting of drug carrier. Comparison with the conventional administration method. Nanomed. Nanotechnol. Biol. Med..

[29] Song X., Fu W., Cheang U.K. (2022). Immunomodulation and delivery of macrophages using nano-smooth drug-loaded magnetic microrobots for dual targeting cancer therapy. iScience.

[30] Karthika V., Alsalhi M.S., Devanesan S., Gopinath K., Arumugam A., Govindarajan M. (2020). Chitosan overlaid Fe_3_O_4_/rGO nanocomposite for targeted drug delivery, imaging, and biomedical applications. Sci. Rep..

[31] Lu H., Xu Y., Qiao R., Lu Z., Wang P., Zhang X., Chen A., Zou L., Wang Z. (2020). A novel clustered spio nanoplatform with enhanced magnetic resonance t_2_ relaxation rate for micro-tumor detection and photothermal synergistic therapy. Nano Res..

[32] Taheri-Kafrani A., Shirzadfar H., Abbasi Kajani A., Kudhair B.K., Jasim Mohammed L., Mohammadi S., Lotfi F. (2021). Functionalized graphene oxide/Fe_3_O_4_ nanocomposite: A biocompatible and robust nanocarrier for targeted delivery and release of anticancer agents. J. Biotechnol..

[33] Mukha I., Chepurna O., Vityuk N., Khodko A., Storozhuk L., Dzhagan V., Zahn D., Ntziachristos V., Chmyrov A., Ohulchanskyy T.Y. (2021). Multifunctional magneto-plasmonic Fe_3_O_4_/Au nanocomposites: Approaching magnetophoretically-enhanced photothermal therapy. Nanomaterials.

[34] Perez-Garnes M., Morales V., Sanz R., Garcia-Munoz R.A. (2021). Cytostatic and cytotoxic effects of hollow-shell mesoporous silica nanoparticles containing magnetic iron oxide. Nanomaterials.

[35] Jesus A.C.B., Jesus J.R., Lima R.J.S., Moura K.O., Almeida J.M.A., Duque J.G.S., Meneses C.T. (2020). Synthesis and magnetic interaction on concentrated Fe_3_O_4_ nanoparticles obtained by the co-precipitation and hydrothermal chemical methods. Ceram. Int..

[36] Ahmed A., Mandal S., Gines L., Williams O.A., Cheng C. (2016). Low temperature catalytic reactivity of nanodiamond in molecular hydrogen. Carbon.

[37] Qiu L., Chen T., Ocsoy I., Yasun E., Wu C., Zhu G., You M., Han D., Jiang J., Yu R. (2015). A cell-targeted, size-photocontrollable, nuclear-uptake nanodrug delivery system for drug-resistant cancer therapy. Nano Lett..

[38] Arami H., Khandhar A., Liggitt D., Krishnan K.M. (2015). In vivo delivery, pharmacokinetics, biodistribution and toxicity of iron oxide nanoparticles. Chem. Soc. Rev..

[39] Yao H., Yan J., Shao P., Wang Y., Liu T., Jiang J., Liu T. (2021). Co-modification with msc membrane and pda prevents Fe_3_O_4_-induced pulmonary toxicity in mice via ampk-ulk1 axis. Toxicol. Lett..

[40] Zhang K., Zhao Q., Qin S., Fu Y., Liu R., Zhi J., Shan C. (2019). Nanodiamonds conjugated upconversion nanoparticles for bio-imaging and drug delivery. J. Colloid Interface Sci..

[41] Dong S., Wang S., Wang X., Zhai L. (2020). Superparamagnetic nanocomposite Fe_3_O_4_@SiO_2_-NH_2_/CQDs as fluorescent probe for copper (ii) detection. Mater. Lett..

