# Supplementary material for: Synthesis of Multi-Stimuli Responsive Fe3O4 Coated with Diamonds Nanocomposite for Magnetic Assisted Chemo-Photothermal Therapy
Source: Molecules. 2023 Feb 13;28(4):1784. doi: 10.3390/molecules28041784 (PMC9959610; doi:10.3390/molecules28041784)
Supplement: Supplementary file 1 [file molecules-28-01784-s001.zip › molecules-2159516-supplementary.pdf]

Supporting Information

Synthesis of Multi-Stimuli Responsive Fe_3O_4 -Coated with Diamonds Nanocomposite for Magnetic Assisted Chemo- photothermal Therapy

Yang Li[1], Ji chuan Kong[1,*], Huan Zhao[2] and Yao Liu[1]

[1] School of Medicine, Henan Polytechnic University, Jiaozuo 454000, China;
[2] Zhengzhou University, Affiliated Hosp 1, Zhengzhou 450000, China
* Correspondence: kongjichuan@hpu.edu.cn(J.K.)

Photothermal conversion efficiency calculation

The photothermal conversion efficiency (η) was calculated ***via*** the following formula :

$$\boldsymbol{\eta = \frac{hS\Delta T_{max} - Q_S}{I(1-10^{-A})} = \frac{hS(\Delta T_{max} - \Delta T_{maxs})}{I(1-10^{-A})}} \qquad \text{(A1)}$$

$$\boldsymbol{hS = \frac{m_s C_S}{\tau}} \qquad \text{(A2)}$$

Where $\triangle T_{max}$ represents the temperature change of sample at the maximumsteady-state temperature; $\triangle T_{maxs}$ represents the temperature change of solvent at themaximum steady-state temperature; I is NIR laser power density; A represents the808 nm absorbance of sample; C_s and m_s are the heat capacity and mass of solvent, respectively; $\boldsymbol{\tau}$ is the sample system time constant, which can be determined by the

linear curve fitting of temperature cooling time *vs* its ln $\frac{\Delta T}{\Delta T_{max}}$(θ =$\frac{\Delta T}{\Delta T_{max}}$).

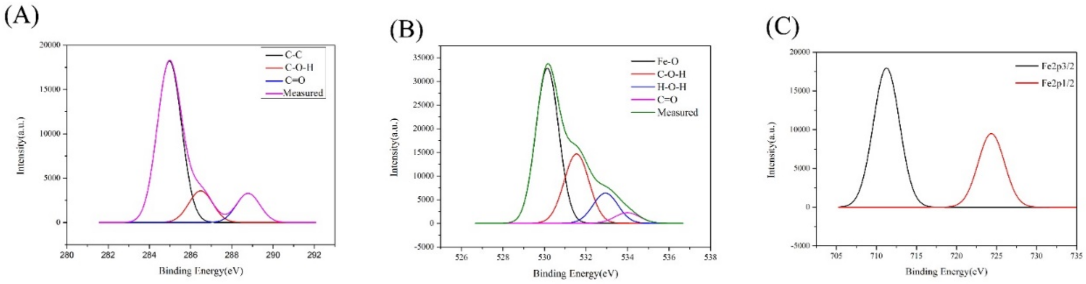

Figure S1. The XPS spectra of C1s (**A**), O1s (**B**), Fe2p (**C**).

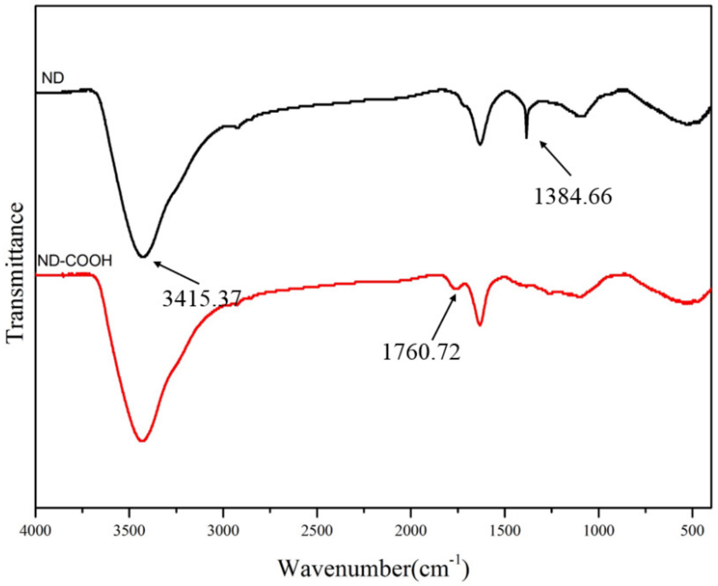

Figure S2. FTIR spectrometry of ND and ND-COOH.

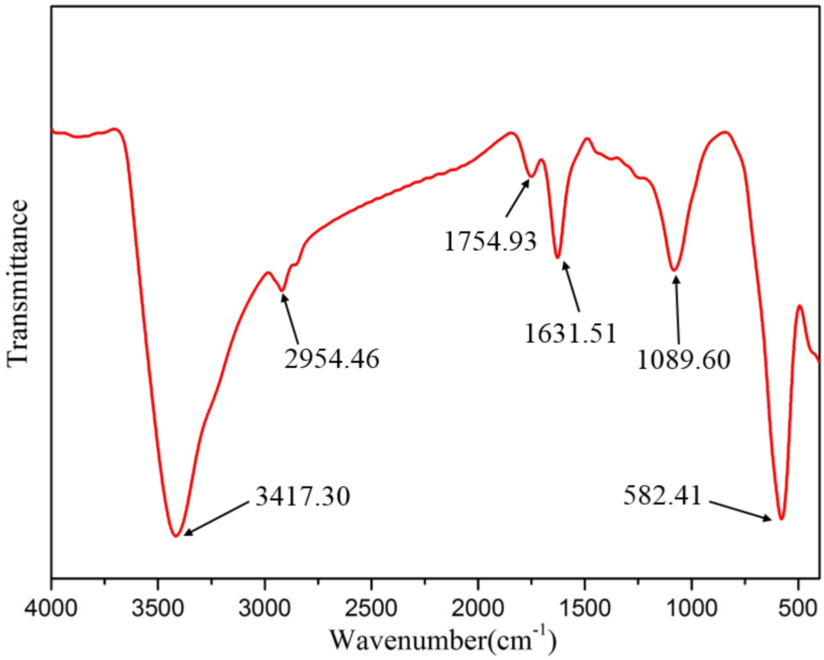

Figure S3. FTIR spectrometry of MNC@ND.

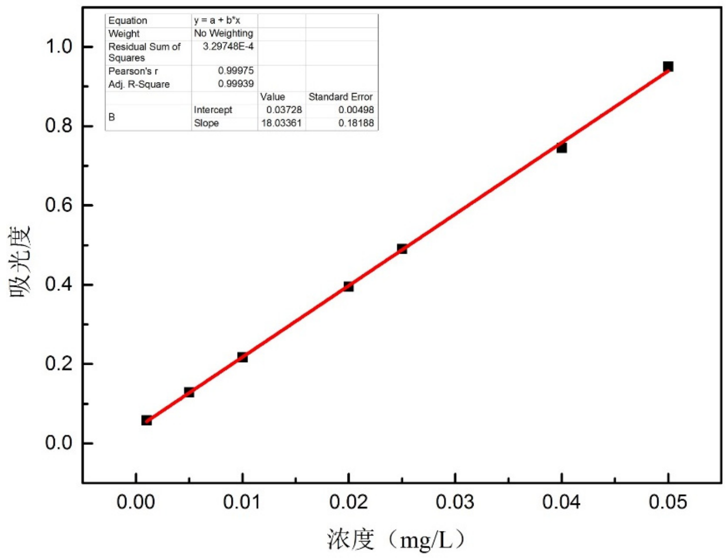

Figure S4. The standard curves of UV-via absorption values of DOX.

The equation of DOX's standard curve is Y=18.03361X+0.03728. TheR^2=0.99939, where X is the concentration of DOX, and Y is the absorbance.
The equation of drug release is[(Y-0.03728)/18.03361]*50/1.6*100

$$A = \frac{V(Y - 0.03728)}{18.03361M} 100\%$$

where A is thedrug release rateof DOX, V is the volume ofdrug release system, M is the mass of the load DOX,and Y is the absorbance.
